# Estimating the effects of physical violence and serious injury on health-related quality of life: Evidence from 19 waves of the Household, Income and Labour Dynamics in Australia Survey

**DOI:** 10.1007/s11136-022-03190-3

**Published:** 2022-08-08

**Authors:** Syed Afroz Keramat, Kim-Huong Nguyen, Francisco Perales, Abdul-Aziz Seidu, Aliu Mohammed, Bright Opoku Ahinkorah, Tracy Comans

**Affiliations:** 1 Faculty of Medicine, Centre for Health Services Research, The University of Queensland, Brisbane, QLD Australia; 2 Economics Discipline, Social Science School, Khulna University, Khulna, Bangladesh; 3 School of Social Science, The University of Queensland, Michie Building (#9), St Lucia, Brisbane, QLD 4067 Australia; 4 Department of Population and Health, University of Cape Coast, Cape Coast, Ghana; 5 School of Public Health, University of Technology Sydney, Sydney, Australia; 6 Global Brain Health Institute, Trinity College Dublin, Dublin, Ireland

**Keywords:** Physical violence, Serious injury, Health-related quality of life, SF-36, Australia

## Abstract

**Objective:**

This study aims to investigate the effect of physical violence and serious injury on health-related quality of life in the Australian adult population.

**Methods:**

This study utilised panel data from the Household, Income and Labour Dynamics in Australia (HILDA) Survey. HRQoL was measured through the physical component summary (PCS), mental component summary (MCS), and short-form six-dimension utility index (SF-6D) of the 36-item Short-Form Health Survey (SF-36). Longitudinal fixed-effect regression models were fitted using 19 waves of the HILDA Survey spanning from 2002 to 2020.

**Results:**

This study found a negative effect of physical violence and serious injury on health-related quality of life. More specifically, Australian adults exposed to physical violence and serious injury exhibited lower levels of health-related quality of life. Who experienced physical violence only had lower MCS (β = −2.786, 95% CI: −3.091, −2.481) and SF-6D (β = −0.0214, 95% CI: −0.0248, −0.0181) scores if switches from not experiencing physical violence and serious injury. Exposed to serious injury had lower PCS (β = −5.103, 95% CI: −5.203, −5.004), MCS (β = −2.363, 95% CI: −2.480, −2.247), and SF-6D (β = −0.0585, 95% CI: −0.0598, −0.0572) score if the adults not experiencing physical violence and serious injury. Further, individuals exposed to both violence and injury had substantially lower PCS (β = -3.60, 95% CI: -4.086, -3.114), MCS (β = −6.027, 95% CI: −6.596, −5.459), and SF-6D (β = −0.0716, 95% CI: −0.0779, −0.0652) scores relative to when the individuals exposed to none.

**Conclusion:**

Our findings indicate that interventions to improve Australian adults’ quality of life should pay particular attention to those who have experienced physical violence and serious injury. Our findings suggest unmet mental health needs for victims of physical violence and serious injuries, which calls for proactive policy interventions that provide psychological and emotional therapy.

## Introduction

Globally, physical violence remains one of the major causes of injuries that result in deaths and disabilities [1, 2]. Despite the adoption of the World Health Organisation’s Resolution WHA49.25 in 1996, which called for the implementation of programmes to prevent the occurrence of violence [3, 4], the global burden of physical violence remains high [2]. In 2016, for instance, interpersonal physical violence resulted in approximately 390,800 deaths across the world [2]. It is currently ranked the 26th cause of disability-adjusted life years (DALYs) worldwide, having declined marginally from the 24th position over the past three decades [2]. Physical violence-related injuries and associated deaths and disabilities are mostly preventable and occur mainly among youth and young adults aged 15–44 years [3, 4]. In Australia, approximately 3.7 million men (4 in 10 men) and 2.9 million women (3 in 10 women) aged 18 years and over have experienced physical violence since age 15 [5]. Additionally, injuries, including interpersonal physical violence-related injuries, are among the top five contributors to DALYs and the national burden of diseases in Australia (Australian Institute of Health and Welfare, 2020). For example, in 2015–2016, injuries accounted for 7.6% (8.9 billion dollars) of the Australian healthcare system’s total healthcare expenditure [6].

Physical violence involves the intentional use of physically aggressive acts such as beating, kicking, and strangling another person or group that could result in injury or have a high tendency to cause injury or death [4, 7]. It is often perpetrated by close acquaintances (e.g. intimate partners, friends, and parents) and is mostly triggered by easy access to weapons and the use of psychotropic drugs and alcohol [8]. Victims of physical violence may suffer serious injuries such as fractures of extremities [9], spinal cord and head injuries [10], which could affect their physical functioning and daily-life activities [11]. Although serious injuries predict poor HRQoL [9, 10], how their severity affects HRQoL remains unclear. An earlier cross-sectional study of severely injured trauma patients found that poor HRQoL was associated with psychosocial factors such as pre-injury co-morbidity, inability to return to work and living alone, but not with the severity of injury [12]. Similarly, a 5-year longitudinal study found that the severity of injuries did not determine the victims’ HRQoL [13]. However, a recent study claimed that poor HRQoL of trauma victims was primarily predicted by factors such as the severity of injury (having more than three days’ intensive care unit stay), type, and location of injury [11].

The available literature on the relationship between injuries and HRQoL has mainly focussed on accidental or unintentional causes such as work-related injuries [14], sports-related injuries [15], and road traffic accidents and falls [9]. Additionally, a few studies have investigated HRQoL among injured domestic violence victims or intimate partner violence [16]. There is limited research examining how serious injuries caused by interpersonal physical violence influence HRQoL. Thus, understanding the relationships between interpersonal physical violence-related serious injuries and HRQoL could help develop tailored interventions at the initial stages of treatment and prioritisation of resources for victims. To fill this gap in research evidence, the present study examined the relationships between physical violence and serious injury with HRQoL in the Australian adult population, using panel data from the Household, Income and Labour Dynamics in Australia (HILDA) Survey. Our results can generate novel and useful insights for policymakers to develop interventions that target the population at risk of physical violence, serious injuries, and poor HRQoL outcomes.

## Methodology

### Data source and sample selection

This study utilised de-identified person data of the Household, Income and Labour Dynamics in Australia (HILDA) Survey. HILDA is Australia’s biggest household-based longitudinal survey and one of the largest in the world [17]. The survey commenced in 2001 following the design of the German Socio-Economic Panel (SOEP), the British Household Panel Survey (BHPS), and the US Panel Study of Income Dynamics (PSID) to establish a nationally representative sample [18]. HILDA collects annual information on diverse domains from more than 13,000 individuals living in over 7,000 households in Australia. Data were collected from individuals aged 15 years or older in each household through personalised interviews (self-completion questionnaires and face-to-face interviews by trained interviewers). Details about the HILDA survey design and procedures have been published elsewhere [18].

**Fig. 1:**
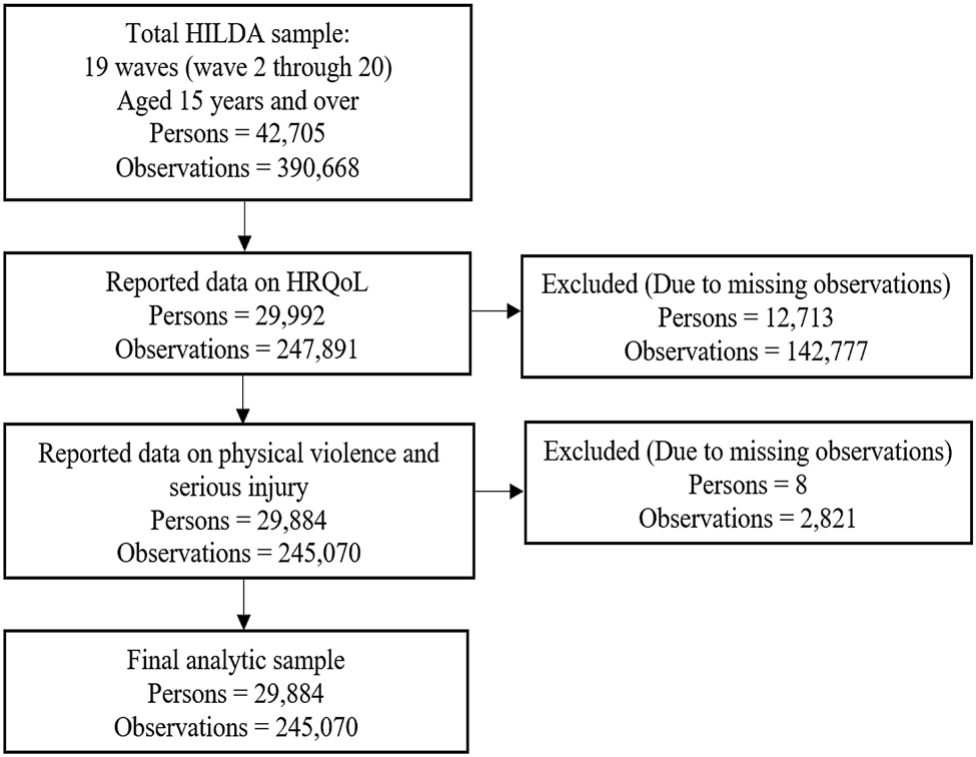
Participants flow into the analytic sample, and missing data

This study utilised the last 19 waves (waves 2 through 20) of the HILDA Survey, spanning the period 2002 to 2020. The main reason for using these waves is that information on key variables of interest (physical violence, serious injury, and markers of HRQoL) was collected in these waves. Observations were excluded if there was missing data on the outcome variable (eight dimensions of the SF-36) and physical violence and serious injury. The final analytic sample consists of 245,070 person-year observations from 29,884 unique participants. Figure 1 shows the step-by-step selection of study participants.

### Outcomes variables

The outcome variable, health-related quality of life (HRQoL), is operationalised through the 36-item Short-Form Health Survey (SF-36). The SF-36 is a broadly used generic, coherent, and easily accomplished questionnaire form to measure an individual’s physical and mental health status [19]. It measures eight health dimensions: physical functioning (PF), role physical (RP), role emotional (RE), social functioning (SF), mental health (MH), vitality (VT), bodily pain (BP), and general health (GH) through 36 questions. The score scale for each dimension of the SF-36 is transformed to a 0–100 range, where 0 indicates the worst health state and 100 indicates the best health state.

The eight dimensions of the SF-36 were further used to derive two distinct higher-ordered composite summary measures: the physical component summary (PCS) and mental component summary (MCS). The PCS is based on four subscales of the SF-36: PF, RP, BP, and GH. The MCS is based on the other four subscales: RE, SF, MH, and VT. Both the PCS and MCS scores were standardised to have a mean of 50 and a standard deviation of 10, respectively. The PCS scores ranged from 4.54 to 76.09 and the MCS scores from −1.21 to 76.19, with higher scores reflecting better quality of life.

The SF-6D is a global index derived from the SF-36 to capture respondents’ health state utility (Norman et al., 2014). This utility index is often used for economic evaluation (to estimate QALY and DALY). The SF-6D score is derived from responses from six dimensions of the SF-36, including PF, RP, RE, SF, VT, and BP. Each of the six dimensions has a level between 2 to 6 that can predict 18,000 health states [20]. The SF-6D score ranges from 0.29 (worst health) to 1 (full health state).

### Exposure variables

Physical violence and serious injury are the primary variables of interest for the current study. Information on both variables was collected in every HILDA Survey wave, except for Wave 1. Data on physical violence were collected by asking respondents whether they had been a victim of physical violence in the past twelve months. Similarly, respondents were asked whether they had experienced any serious injury or illness to self in the previous twelve months. Responses to both questions were taken in binary form (0 = ‘no’; 1 = ‘yes’). The authors utilised these two variables to construct a new variable, ‘physical violence and serious injury’. The main exposure was categorised as ‘none’, ‘no violence, but injury’, ‘violence, but no injury’, and ‘both violence and injury’.

### Control variables

A number of control variables were included in the statistical analyses to account for potential confounding. Following existing studies on HRQoL [19, 21, 22], this study included a compassing set of factors known to be correlated with HRQoL. These are age, gender, relationship status, highest education level completed, household annual disposable income, labour force status, Indigenous status, region of residence, smoking status, alcohol consumption, and physical activity. Descriptions of all covariates used in the analysis are provided in Table 1.

**Table 1:** Description of the control variables

Name of the control	Category
Age	15–24, 25–39, 40–64, and ≥ 65 years
Gender	Male and female
Relationship status	Single (never married and not living with someone, separated, divorced, and widowed), and couple (married, and living with someone)
Highest education level completed	Year 12 and below (year 12, and ≤ year 11), certificate courses (Diploma, and certificate III/IV), and university degrees (masters or doctorate, graduate diploma, and honours)
Household annual disposable income	Quintile 1 (poorest), quintile 2 (poorer), quintile 3 (middle), quintile 4 (richer), and quintile 5 (richest)
Labour force status	Employed, and unemployed or not in the labour force (NLF)
Indigenous status	Not of Indigenous origin, and Indigenous origin (Aboriginal, Torres Strait Islander, and/or both)
Region of residence	Major city, and regional or remote area (inner, outer, remote, and very remote Australia)
Smoking status	Never smoked, former smoker, and current smoker (smoke daily, weekly, less than weekly)
Alcohol consumption	Never drunk, ex-drinker, current drinker (only rarely, 1–2, 2–3, 3–4, 5–6, 7 days per week)
Physical activity	Less than the recommended level (not at all, < 1, 1–2 times, and 3 times moderate or intensive physical activity for at least 30 minutes a week), and recommended level (> 3 times a week, and every day)

### Estimation strategy

An unbalanced longitudinal dataset consisting of 245,070 person-year observations was constructed by linking 29,884 de-identified records from individuals who participated in at least one survey wave (from waves 2 through 20). To summarise the characteristics of the study participants, descriptive statistics in the form of frequency (n) and percentage (%) were reported for categorical variables. The mean along with the standard deviation (SD) was reported for continuous variables.

Our main analyses involve multivariate regression models estimating the relationships between physical violence and injury and HRQoL. We fitted three different models for continuous dependent variables: PCS, MCS, and SF-6D. This study utilised fixed-effects longitudinal regression model to estimate the effect of physical violence and serious injury on HRQoL. The model estimates how switches from individuals’ exposure to physical violence and serious injury are associated with deviations from their usual outcomes (captured by the individual mean scores in HRQoL over time). It takes the following form:

1 $${HRQoL}_{it}- \overline{{HRQoL}_{i}} = {\beta }_{1}{ (PVSA}_{it}- \overline{{PVSA}_{i}})+ {\beta }_{2}{ (X}_{it}- \overline{{X}_{i}}) + ({\varepsilon }_{it}- \overline{{\varepsilon }_{i}})$$

In Eq. 1, ${HRQoL}_{it}$ refers to the summary measures (PCS and MCS), and the health utility index (SF-6D). PVSA is the main variable of interest that captures physical violence and serious injury experienced by the respondents. X is a vector of control variables, ${\varepsilon }_{it}$ is the error term, subscripts i refer to individual and t indicates time.

Fixed-effects model captures the underlying reasons for variations in outcomes within a person across different periods. In the current study, the fixed-effects models estimate how within-person variations in a person’s HRQoL differ in those observation periods in which they were exposed to physical violence and injury compared to those in which they were not exposed to physical violence and serious injury. All models were adjusted for the socio-demographic and lifestyle characteristics described before. This study considers statistical significance for the exposure variables at *p* < 0.05. All analyses were conducted using Stata version 16.0 (Stata SE 16, Stata Corp, College Station, TX, USA).

## Results

### Descriptive statistics

A summary of socio-economic and health-related characteristics of the study sample at baseline, final, and all waves pooled is presented in Table 2. Among the study participants, over one-third (41%) were aged 46–64 years, more than half (53%) were female, and approximately 60% were coupled, one-fourth (25%) have university degrees, two-thirds (65%) are employed, are non-Indigenous (97%), and nearly two-third (65.73%) lives in major cities. Variations also existed concerning the study participants’ health-related characteristics. More than half (54%) of the study participants never smoked, a vast proportion (82%) consumed alcohol, and two-thirds (66%) did not engage in the recommended level of physical activity (pooled sample).

**Table 2:** Distribution of the analytic sample (socio-demographic and health-related characteristics): Baseline, final wave, and all waves pooled (unique persons = 29,884; person-year observations = 245,070)

Characteristics	Baseline wave (2002)					Final wave (2020)				Pooled sample (2002 through 2020)		
	n		%			n		%		n		%
Socio-demographic characteristics												
Age												
15–24 years	1803		16.53			2152		14.82		42,090		17.17
25–39 years	3182		29.17			4144		28.53		63,405		25.87
40–64 years	4533		41.56			5380		37.04		99,502		40.60
≥ 65 years	1389		12.73			2847		19.60		40,073		16.35
Gender												
Male	5190		47.58			6657		45.84		114,987		46.92
Female	5717		52.42			7866		54.16		130,083		53.08
Relationship status												
Single	4246		38.93			5769		39.72		98,125		40.04
Couple	6661		61.07			8754		60.28		146,945		59.96
Highest education level completed												
Year 12 and below	5889		53.99			5446		37.50		110,024		44.89
Certificate courses	2875		26.36			4797		33.03		74,581		30.43
University degrees	2143		19.65			4280		29.47		60,465		24.67
Household annual disposable income												
Quintile 1 (Poorest)	2182		20.01			2905		20		49,016		20.00
Quintile 2	2181		20			2905		20		49,012		20.00
Quintile 3	2185		20.03			2904		20		49,015		20.00
Quintile 4	2179		19.98			2906		20.01		49,016		20.00
Quintile 5 (Richest)	2180		19.99			2903		19.99		49,011		20.00
Labour force status												
Employed	7031		64.46			9132		62.88		158,737		64.77
Unemployed or NLF	3876		35.54			5391		37.12		86,333		35.23
Indigenous status												
Not of Indigenous origin	10,653		97.68			13,995		96.36		237,802		97.04
Indigenous origin	253		2.32			528		3.64		7,265		2.96
Region of residence												
Major city	6936		63.59			9576		65.94		161,073		65.73
Regional or remote area	3971		36.41			4947		34.06		83,997		34.27
Health-related characteristics												
Smoking status												
Never smoked	5318		48.76			8348		57.48		131,974		53.85
Ex-smoker	2944		26.99			3948		27.18		66,689		27.21
Current smoker	2645		24.25			2227		15.33		46,407		18.94
Alcohol consumption												
Never drunk	1134		10.40			1491		10.27		26,174		10.68
Ex-drinker	637		5.84			1388		9.56		18,543		7.57
Current drinker	9136		83.76			11,644		80.18		200,353		81.75
Physical activity												
Less than the recommended level	7167		65.71			9484		65.30		162,316		66.23
Recommended level	3740		34.29			5039		34.70		82,754		33.77

Summary statistics of the subjective health scores of the analytic sample as well as the status of physical violence and serious injury are shown in Table 3. Mean scores on each of the eight SF-36 domains are PF (83.84 ± 23.00), RP (79.00 ± 36.02), RE (82.44 ± 33.37), SF (82.30 ± 23.50), MH (73.78 ± 17.38), VT (59.45 ± 19.96), BP (73.01 ± 23.92), and GH (68.04 ± 20.91). The mean SF-36 component summary measures (PCS and MCS) and health utility index (SF-6D) are 49.45 ± 10.38, 48.38 ± 10.64, 0.76 ± 0.12, respectively (pooled in all waves). Table 3 also shows that 90% of the sample did not experience physical violence or serious injury. Concerning the key exposure variable, 8.4% of adults had no injury but serious illness, 1.15% had experienced violence but no injury, and 0.34% had experienced both (pooled sample).

**Table 3:** Distribution of subjective health scores of the analytic sample along with their physical violence and serious injury status: Baseline, final wave, and all waves pooled (unique persons = 29,884; person-year observations = 245,070)

Variables	Baseline wave (2002)		Final wave (2020)		Pooled sample (2002 through 2020)	
	n	Mean (SD)	n	Mean (SD)	n	Mean (SD)
SF-36 domain scores						
Physical functioning	10,907	83.91 (22.45)	14,523	84.33 (22.75)	245,070	83.84 (23.00)
Role physical	10,907	80.17 (34.71)	14,523	76.80 (36.96)	245,070	79.00 (36.02)
Role emotional	10,907	83.61 (31.91)	14,523	75.68 (37.46)	245,070	82.44 (33.37)
Social functioning	10,907	82.80 (23.12)	14,523	80.07 (24.49)	245,070	82.30 (23.50)
Mental health	10,907	74.36 (17.10)	14,523	70.71 (18.42)	245,070	73.78 (17.38)
Vitality	10,907	60.87 (19.78)	14,523	56.40 (20.74)	245,070	59.45 (19.96)
Bodily pain	10,907	75.07 (24.01)	14,523	71.94 (23.48)	245,070	73.01 (23.92)
General health	10,907	69.89 (20.90)	14,523	66.48 (20.48)	245,070	68.04 (20.91)
SF-36 component summary scores						
PCS	10,907	49.91 (10.15)	14,523	49.86 (10.60)	245,070	49.45 (10.38)
MCS	10,907	48.82 (10.31)	14,523	46.10 (11.55)	245,070	48.38 (10.64)
Utility score						
SF-6D	10,907	0.76 (0.12)	14,523	0.74 (0.12)	245,070	0.76 (0.12)
Physical violence and serious injury, n (%)						
None	9,823	90.06	13,112	90.28	220,744	90.07
No violence but injury	857	7.86	1,178	8.11	20,676	8.44
Violence but no injury	178	1.63	172	1.18	2,819	1.15
Both violence and injury	49	0.45	61	0.42	831	0.34

**Fig. 2:**
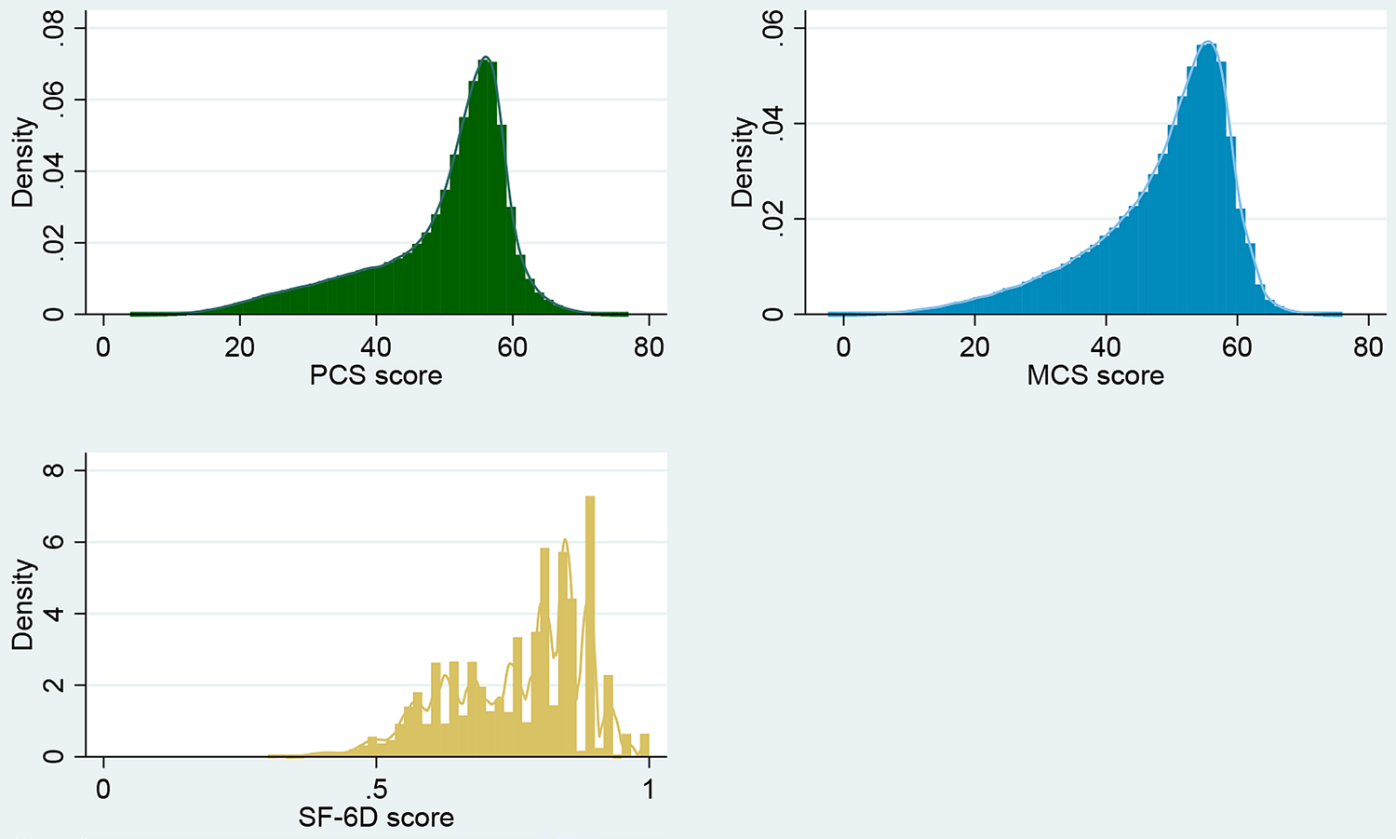
Distribution of PCS and MCS scores and SF-6D utility values

The distribution of the outcome variables (PCS, MCS and SF-6D utility score) is shown in Fig. 2. The figure reveals that most respondents had PCS and MCS scores between 50 and 60. The SF-6D score is skewed to the right, with a lot of observations having a score greater than 0.7. There are a relatively small number of observations with a score of 0.4 to 0.6.

**Fig. 3:**
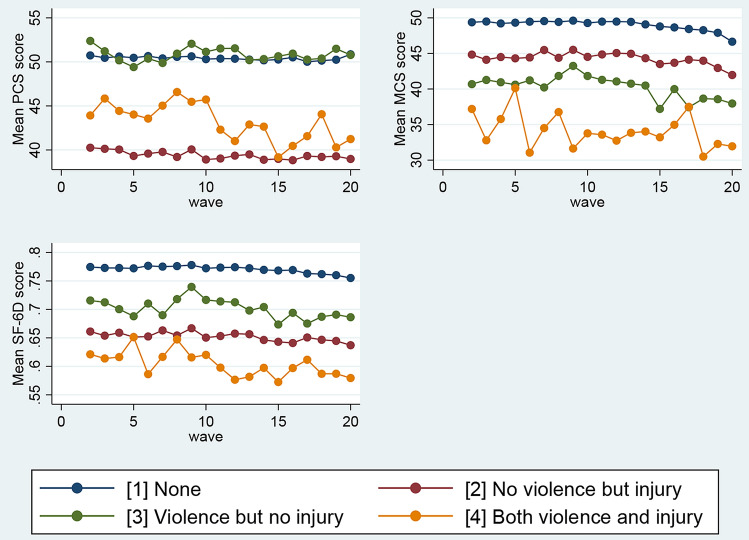
Mean SF-36 component summary scores and SF-6D utility score by physical violence and serious injury, waves 2–20

Figure 3 displays the mean PCS, MCS, and SF-6D scores by physical violence and serious injury. The figure demonstrates that mean PCS, MCS, and SF-6D scores decline for participants exposed to either physical violence, serious injury, or both. Participants who had been exposed to both physical violence and serious injury had lower mean PCS, MCS, and SF-6D scores. For example, mean PCS, MCS, and SF-6D scores in Wave 20 were much lower among the respondents exposed to both physical violence and serious injury (41.24, 39.95, and 0.58, respectively) compared to participants exposed to none (50.86, 46.65, and 0.76, respectively).

### Regression modelling

Table 4 presents the result from the fixed-effects regression models examining the combined effects of physical violence and serious injury on HRQoL measured through PCS, MCS, and SF-6D. Model 1 indicates that participants reported lower PCS scores in those observations in which they had been exposed to serious injury (β = −5.103) or both physical violence and injury (β = −3.60) than in those observations in which they had been exposed to neither of these stressors. However, a statistically positive significant difference in PCS score has been found in terms of experiencing physical violence (0.376). Model 2 also shows that adults exposed to only injury (−2.363), only physical violence (−2.786) and both (−6.027) had lower MCS scores if switched from not experiencing physical violence and serious injury.

**Table 4:** The relationship between physical violence and serious injury with the SF-36 component summary scores and SF-6D utility score, longitudinal fixed-effects regression

Variables	Model 1: PCS	Model 2: MCS	Model 3: SF-6D
	β [95% CI]	β [95% CI]	β [95% CI]
Physical violence and serious injury			
None (ref)			
No violence but injury	−5.103^***^ [−5.203, −5.004]	−2.363^***^ [−2.480, −2.247]	−0.0585^***^ [−0.0598, −0.0572]
Violence but no injury	0.376^**^ [0.115, 0.636]	−2.786^***^ [−3.091, −2.481]	−0.0214^***^ [−0.0248, −0.0181]
Both violence and injury	−3.60^***^ [−4.086, −3.114]	−6.027^***^ [−6.596, −5.459]	−0.0716^***^ [−0.0779, −0.0652]
Age			
15–24 years (ref)			
25–39 years	0.215^**^ [0.0786, 0.352]	−1.653^***^ [−1.812, −1.493]	−0.0090^***^ [−0.0108, −0.0073]
40–64 years	−0.967^***^ [−1.152, −0.782]	−2.226^***^ [−2.442, −2.010]	−0.0214^***^ [−0.0238, −0.0190]
≥ 65 years	−3.181^***^ [−3.411, −2.950]	−1.494^***^ [−1.764, −1.224]	−0.0292^***^ [−0.0322, −0.0262]
Gender			
Male			
Female			
Relationship status			
Single			
Couple	−0.0423 [−0.148, 0.063]	0.865^***^ [0.742,0.989]	0.0084^***^ [0.0070, 0.0097]
Highest education level completed			
Year 12 and below			
Certificate courses	−0.558^***^ [−0.728, −0.387]	−0.436^***^ [−0.635, −0.236]	−0.0077^***^ [−0.0099, −0.0055]
University degrees	−0.667^***^ [−0.877, −0.457]	−0.543^***^ [−0.788, −0.297]	−0.0101^***^ [−0.0128, −0.0073]
Household annual disposable income			
Quintile 1 (Poorest)	1.291^***^ [1.169, 1.413]	−0.390^***^ [−0.533, −0.247]	0.0052^***^ [0.0037, 0.0068]
Quintile 2	0.705^***^ [0.596, 0.814]	−0.395^***^ [−0.522, −0.268]	0.002^**^ [0.0006, 0.0034]
Quintile 3	0.508^***^ [0.408, 0.608]	−0.192^**^ [−0.309, −0.0753]	0.0023^***^ [0.001, 0.0036]
Quintile 4	0.222^***^ [0.130, 0.313]	−0.0573 [−0.165,0.0502]	0.0012^*^ [0.0001, 0.0024]
Quintile 5 (Richest) (ref)			
Labour force status			
Employed (ref)			
Unemployed or NLF	−1.311^***^ [−1.398, −1.224]	−0.807^***^ [−0.909, −0.705]	−0.0167^***^ [−0.0178, −0.0155]
Indigenous status			
Not of Indigenous origin			
Indigenous origin			
Region of residence			
Major city (ref)			
Regional or remote area	−0.169^*^ [−0.313, −0.0246]	0.174^*^ [0.00552,0.343]	−0.0002 [−0.0021, 0.0016]
Smoking status			
Never smoked (ref)			
Ex-smoker	−0.0967 [−0.250,0.0563]	−0.553^***^ [−0.732, −0.374]	−0.006^***^ [−0.008, −0.004]
Current smoker	0.524^***^ [0.350,0.697]	−1.390^***^ [−1.594, −1.187]	−0.0083^***^ [−0.0106, −0.0061]
Alcohol consumption			
Never drunk (ref)			
Ex-drinker	−0.482^***^ [−0.657, −0.306]	−1.422^***^ [−1.627, −1.217]	−0.0151^***^ [−0.0174, −0.0128]
Current drinker	1.045^***^ [0.900, 1.191]	−1.098^***^ [−1.268, −0.927]	−0.0026^**^ [−0.0045, −0.0007]
Physical activity			
Less than the recommended level (ref)			
Recommended level	1.231^***^ [1.163, 1.299]	1.584^***^ [1.505, 1.663]	0.0215^***^ [0.0206, 0.0224]

95% confidence intervals in brackets

*p < 0.05, **p < 0.01, ***p < 0.001

*ref* reference category, *PCS* Physical Component Summary, *MCS* Mental Component Summary, *SF-6D* Short-Form Six-Dimension health index

Statistically significant lower scores in SF-6D score are also observed among adults exposed to physical violence and serious injury compared with those exposed to none. Adults exposed to physical violence (−0.0585), serious injury (−0.0214), and both (−0.0716) had lower SF-6D scores if the adults were exposed to none (Model 3).

## Discussion

This study aimed to examine the association between physical violence, serious injuries, and HRQoL in the Australian adult population. The analysis was based on 19 consecutive waves of longitudinal data from the HILDA Survey spanning the 2002 to 2020 period. The results showed that 1.15% and 8.44% of the respondents had experienced physical violence and serious injury, respectively. The main findings suggest that exposure to physical violence and serious injury were associated with a reduction in HRQoL as measured by the SF-36. Specifically, of the summary measures and health utility index of SF-36 (PCS, MCS and SF-6D), victims of only physical violence have reduced MCS and SF-6D utility scores. Similarly, individuals exposed to only serious injuries and both physical violence and serious injuries scored substantially lower for PCS, MCS, and SF-6D. These associations were statistically significant even after controlling for age, gender, relationship status, highest education level completed, household annual disposable, labour force status, Indigenous status, region of residence, smoking status, alcohol consumption, and physical activity.

The negative associations between MCS and SF-6D with physical violence found in the present study were consistent with those reported in the previous literature [23, 24]. The emotional problems associated with experiencing physical violence could plausibly account for the poor mental health outcomes (low MCS scores) among the victims compared to other adults. For instance, an earlier study reported that victims of physical violence experience long-term adverse mental health consequences that negatively impact their quality of life [25]. This might have probably also contributed to the worst possible health state (low SF-6D) recorded among victims of physical violence compared to non-victims.

We found a positive association between physical violence and PCS, which contradicts previous studies [23, 24, 26]. Arguably, victims of physical violence may recover from any related physical health consequences within a relatively shorter duration than the emotional health consequences. However, they may still suffer emotionally, which could negatively affect their mental health, as manifested in the low MCS scores recorded among the victims. This calls for increased psychosocial interventions such as social support for victims of physical violence [27], given that social support improves mental health outcomes among victims of physical violence [25].

The findings from this study also suggest that victims of serious injuries have lower scores for PCS, MCS and SF-6D compared to their peers without serious injuries. Similarly, previous studies had reported a negative association between serious injuries and the PCS [28] and MCS [29]. To the best of our knowledge, the present study is the first that reports an association between serious injury and the SF-6D utility score. Thus, the present study finding is important because it could serve as a reference for future studies on the association between serious injuries and HRQoL as measured by the SF-6D utility score.

The significant negative association between serious injuries and lower PCS and MCS scores, as found in this study, suggests that injured victims may have a worse physical and mental health-related quality of life than those without injuries. Contrary to the findings from the present study, a recent study reported no association between serious injuries and PCS scores, even though the researchers found a significant negative association between serious injuries and MCS scores [29]. Perhaps, the duration of their study (data were collected from road traffic accident victims at 6, 12, and 24 months) might not have been long enough to elicit a significant perception of a decrease in quality of life [29]. Besides, the method, focal population, and type of likely injury (traffic-related) are plausible reasons. Relatedly, another study reported that injury severity was not significantly associated with both PCS and MCS scores [30]. However, the researchers noted that persons with traumatic injuries generally had low PCS and MCS scores. In their prospective study, data were collected at one month and six months post injury, a duration that might be too short to produce any significant association between injury and HRQoL. However, corroborating the findings from the current study, a recent study found a significant association between serious injuries and low PCS among victims of the 9/11 World Trade Center attack in the US, fifteen years after the incident [27].

This study’s findings add to increasing evidence indicating that physical violence and serious injuries are associated with reduced HRQoL. Given that most previous studies used the SF-36 (a general measure of HRQoL) in examining the relationship between HRQoL and physical violence [31, 32] or serious injuries [10, 33], the current study innovates with respect to the existing literature by using the SF-6D utility score, a generic preference-based measure of HRQoL, which is highly generalisable and can be used to estimate quality-adjusted life years [QALYs] [34, 35]. Additionally, the utilisation of a large sample size helps to get more precise estimates when investigating the relationships between HRQoL and physical violence and serious injuries in the Australian population. Further, to minimise the risk of omitted variable bias, this study employed fixed-effects analytical approach. For example, it is possible that individuals who have certain unobserved characteristics (e.g. housing instability, certain personality types, etc.) tend to attract both violence and poor HRQoL. Fixed-effect models would minimise the possibility of this type of omitted variable bias that affects results.

The present study findings highlight a significant impact of physical violence and serious injuries on HRQoL, especially concerning the mental health states of victims, even in the absence of any form of physical health issues. Thus, there is a possibility of unmet mental health needs for victims of physical violence and serious injuries, which calls for proactive policy interventions that provide psychological and emotional therapy for victims. Therefore, this study recommends that victims of physical violence and serious injuries be screened for mental health problems by healthcare professionals during their first hospital visits with follow-up mental health assessments. Hence, all healthcare professionals in acute care settings should be given regular training on primary mental health screening or assessment skills. All screened patients should be referred to a clinical psychologist for follow-up assessments and management when appropriate. This approach could minimise the long-term mental health impact of physical violence and serious injuries on the Australian population.

This study’s major strength is using a large and nationally representative longitudinal dataset with numerous observations over prolonged periods (19 waves spanning from 2002–2020). Thus, the study findings could be generalisable to victims of physical violence and serious injuries in Australia.

Despite the importance of our findings, it is worthy to note some of the limitations of this study. First, even though the study used longitudinal data, we cannot make causal inferences due to the study’s observational nature. A second limitation of this study is that the sources and type of injuries were not identified in the data. Different sources and types of injuries may impact victims’ quality of life differently, pointing to the need to replicate our analyses using data containing more granular information [9]. Finally, this study—as well as earlier studies—has not considered the longitudinal persistence of the negative impacts of physical violence and serious injuries on HRQoL. Understanding the duration of these negative effects would be helpful in designing remedial interventions, and may also contribute to reconciling the mixed findings from earlier studies described before. Therefore, future research adopting a life-course approach to examine the duration of the negative effects of physical violence and serious injuries on HRQoL is warranted.

## Conclusion

This study has provided novel empirical evidence on the associations between physical violence and serious injuries on HRQoL among adults in Australia. Physical violence and serious injuries were associated with decreased mental health (MCS) and worse health states (SF-6D), while serious injuries were related to decreased physical health (PCS). Thus, there is a possibility of unmet mental health needs for victims of physical violence and serious injuries. This calls for proactive policy interventions that provide psychological and emotional therapy.

## Data Availability

The data used for the study were collected from the Melbourne Institute of Applied Economic and Social Research. There are some restrictions on these data and are not available to the public. Those interested in accessing these data should contact the Melbourne Institute of Applied Economic and Social Research, The University of Melbourne, VIC 3010, Australia.

## Abbreviations

BMI: Body Mass Index
HILDA: Household, Income and Labour Dynamics in Australia Survey
HRQoL: Health-related Quality of Life
PCS: Physical Component Summary
MCS: Mental Component Summary
SF-6D: Short-Form Six-Dimension
SF-36: 36-Item Short-Form Health Survey
